# Blood Pressure Control Among Diabetic Patients in the Eastern Mediterranean Region: A Systematic Review and Meta-Analysis

**DOI:** 10.2174/0115733998327293240729080250

**Published:** 2024-07-31

**Authors:** Amir Hossein Behnoush, Sepehr Khosravi, Fateme Ziamanesh, Rasha Atlasi, Ali Sheidaei, Negin Sanadgol, Amirmohammad Khalaji, Ozra Tabatabaei-Malazy, Afshin Ostovar

**Affiliations:** 1 Non-Communicable Diseases Research Center, Endocrinology and Metabolism Population Sciences Institute, Tehran University of Medical Sciences, Tehran, Iran;; 2 School of Medicine, Tehran University of Medical Sciences, Tehran, Iran;; 3 Tehran Heart Center, Tehran University of Medical Sciences, Tehran, Iran;; 4 Department of Epidemiology and Biostatics, School of Public Health, Tehran University of Medical Sciences, Tehran, Iran;; 5 Endocrinology and Metabolism Research Center, Endocrinology and Metabolism Clinical Sciences Institute, Tehran University of Medical Sciences, Tehran, Iran;; 6 Osteoporosis Research Center, Endocrinology and Metabolism Clinical Sciences Institute, Tehran University of Medical Sciences, Tehran, Iran

**Keywords:** Diabetes, hypertension, blood pressure, health policy, health promotion, chronic metabolic disorder

## Abstract

**Background:**

The control of blood pressure (BP) is a challenge in diabetic patients and is associated with adverse outcomes of diabetes. In this systematic review and meta-analysis, we investigated the BP control rate among hypertensive diabetic patients in the Eastern Mediterranean Region (EMR) countries.

**Methods:**

We systematically searched PubMed, Scopus, Embase, Cochrane, and Web of Science databases up to January 2023 for observational studies on BP control among hypertensive diabetic patients in all EMR countries. We included studies reporting the proportion of hypertensive, type 2 diabetic patients with controlled BP, defined as systolic/diastolic BP < 140/90 or <130/80 mmHg. Study quality was assessed using modified STROBE guidelines, and a random-effect meta-analysis was conducted to pool prevalence data and calculate overall rates. Subgroup analysis was performed by gender, study design, country, and BP control cut-offs (140/90 and 130/80).

**Results:**

Among the 1949 retrieved studies, 20 studies assessing 27956 individuals were included. The proportion of BP control regardless of cut-off points was 36.8% (95% CI=29.1%45.3%) based on the studies reported for both genders. The prevalence was 53.2% (95% CI=36.1%-69.6%) and 43.5% (95% CI=20.0%-70.3%) based on the studies reported just for women or men, respectively.

**Conclusion:**

Our findings indicate that BP control targets are not successfully achieved in hypertensive diabetic patients in the Eastern Mediterranean region. It is recommended to place greater emphasis on the quality of hypertension care in the management of type 2 diabetes.

## 1 INTRODUCTION

Diabetes mellitus, a complex chronic metabolic disorder, poses a significant global health challenge. Notably, the incidence of this condition is steadily increasing due to factors such as the growing elderly and urban populations, the prevalence of obesity, and the trend toward a sedentary lifestyle [1]. This increase is particularly pronounced in developing countries, with 1.31 billion in 2050 [2]. The Eastern Mediterranean (EMR) Region contains a diverse group of developing countries with varying levels of healthcare infrastructure and resources; within this region, diabetes has emerged as a formidable public health concern, with 73 million involved patients and an estimated 95 million in 2030 [3]. One of the critical complications associated with diabetes is hypertension, as it significantly elevates the risk of cardiovascular events and other related complications [4]. Achieving optimal blood pressure of 130/80 mm Hg, recommended by the ADA (American Diabetic Association), is crucial for diabetic patients, as it mitigates the risk of diabetic microvascular and macrovascular complications. Findings of the United Kingdom Prospective Diabetes Study (UKPDS) 33 indicate a 44% reduction in the risk of cerebral vascular accidents, a 32% decrease in mortality, and a 34% decrease in retinopathy [5]. Notably, this study also observed a linear correlation between blood pressure control and the reduction of diabetic complications, ultimately contributing to improved overall health outcomes. The World Health Organization (WHO) has highlighted countries in the Eastern Mediterranean Region (EMR) as areas of concern regarding diabetes management and related comorbidities, such as hypertension. Consequently, there is a pressing need for an in-depth understanding of the prevalence of controlled blood pressure among diabetic patients in this region. Previous cross-sectional studies have reported a wide range in the prevalence of controlled blood pressure (BP) in the EMR, varying from 10% to 71% [6-8]. To provide a comprehensive overview and assess the prevalence of controlled BP among hypertensive diabetic patients in EMR countries, this systematic review and meta-analysis have been conducted. It aims to fill the existing knowledge gap by systematically gathering, evaluating, and synthesizing the relevant literature on this topic. This study will provide valuable insights into hypertension management in diabetic populations within this diverse and dynamic region. Such insights are critical for informing healthcare policies and interventions to enhance the quality of care and health outcomes for individuals with diabetes in the EMR region.

## 2 MATERIALS AND METHODS

**Fig. (1):**
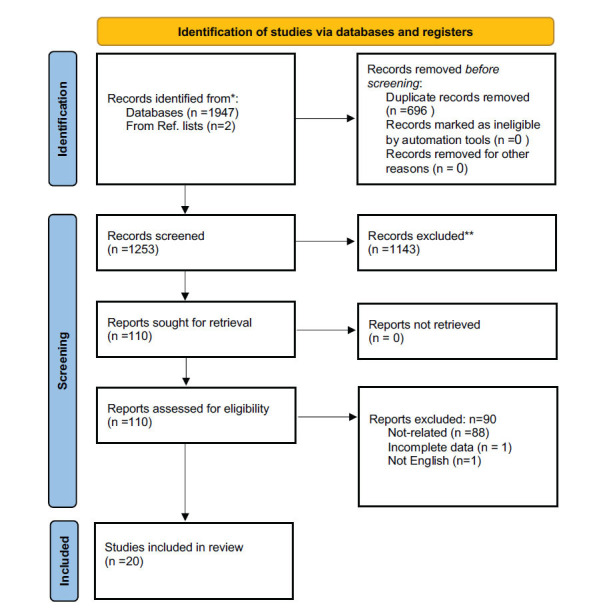
PRISMA flow diagram for search and screening process.

The current systematic review and meta-analysis were performed in accordance with the Preferred Reporting Item for Systematic Reviews and Meta-Analysis (PRISMA) 2020 recommendations [9] (Fig. **1**). We registered the protocol of this systematic review in the International Prospective Register of Systematic Reviews (PROSPERO) with Registration Number CRD42023408342. In addition, the ethics committee of the Endocrinology and Metabolism Research Institute of Tehran University of Medical Sciences has authorized this study (IR.TUMS.EMRI.REC.1401.101).

### 2.1 Search Strategy

International databases, including PubMed, Scopus, Embase, Cochrane Library, and Web of Science, were systematically searched for relevant studies published in English up to January 2023. The used search terms included: “prevalence,” “diabetes, “hypertension,” and “control,” in addition to all relevant MeSH terms in the EMR region and its countries. In addition, the reference lists of all relevant articles were screened to find any eligible studies that might have been missed. Details of the search strategy and the keywords used are presented in Supplementary Table **S1**.

### 2.2 Inclusion/Exclusion Criteria and Study Selection

Studies were included if they investigated the rate of controlled hypertension in hypertensive patients diagnosed with diabetes in EMR region countries, including Afghanistan, Bahrain, Djibouti, Egypt, Iran, Iraq, Jordan, Lebanon, Libya, Morocco, Palestine, Oman, Pakistan, Qatar, Saudi Arabia, Somalia, Sudan, Syrian Arab Republic, Tunisia, United Arab Emirates, and Yemen [10]. Review articles, case reports, case series, and animal studies were excluded. All search results were imported into EndNote 21 software (Thomson Reuters, New York, USA). Following this, two authors independently screened titles and abstracts, adhering to predefined inclusion and exclusion criteria. Any disagreements encountered during this stage were resolved through discussion with a senior third author. Subsequently, these two authors conducted full-text screening, with any discrepancies again being addressed through consultation with the senior third author, thereby completing the screening process.

### 2.3 Data Extraction and Quality Assessment

Data extraction was performed independently by two authors who resolved disagreement through consensus and discussion with a senior third author. The extracted data include the first author’s name, year of publication, country of origin, sample size, mean age, gender, type of diabetes, BP control cutoff, and the rate of controlled BP in hypertensive diabetic patients. To ensure the quality of these included studies, the modified STROBE guidelines were used for their quality assessment.

### 2.4 Statistical Analysis

Statistical analysis was conducted using R software [version 4.3.0]. Random-effect meta-analysis was performed to pool the prevalence of controlled BP and its 95% confidence intervals (CI). Cochrane’s Q test and the I^2^ statistic were used to evaluate inter-study heterogeneity. I^2^ > 50% or *P* < 0.1 reflected the presence of significant heterogeneity [11]. For assessment of each individual’s effect on the overall result, sensitivity analysis by leave-one-out method was performed. Subgroup analysis based on gender, design of studies, target BP, and country was performed, and the difference between the groups was assessed by the Chi-Squared test. Publication bias was evaluated by visually examining funnel plots for asymmetry and Egger’s statistical test [12]. *P* < 0.05 was considered statistically significant throughout the analyses.

## 3 RESULTS

### 3.1 Search and Included Studies Characteristics

A total of 1947 records were obtained from web database searching and two from citation searching, among which 696 were duplicates and removed. The remaining 1253 studies underwent title/abstract screening, and 1143 studies were excluded. The full text of 110 articles was screened in terms of eligibility criteria, of which 90 were excluded due to irrelevance to the topic, incomplete data, and languages other than English. Finally, 20 studies remained to be included in the meta-analysis [7, 8, 13-30]. The PRISMA flowchart summarizes the search and study selection process, which is illustrated in Fig. (**1**). The baseline characteristics of the 20 included studies are mentioned in Table **1**. All studies were conducted on type 2 diabetes mellitus, except three studies conducted on both type 1 and 2 diabetes mellitus [8, 24, 25]. Among countries in the EMR region, three studies were performed in each country: Iran [21, 24, 25], Saudi Arabia [18, 20, 23], Oman [15, 16, 22], Pakistan [13, 14, 19], and Jordan [26, 27, 29], while these studies were conducted from 2002 to 2023. A total of 27956 diabetic individuals with hypertension were investigated in these studies. The cutoff used for hypertension control was 140/90 mmHg in 14 of the studies. Except for Mohammad *et al.*’s [26] study, all studies were deemed high quality according to the STROBE guidelines criteria.

### 3.2 Meta-analysis

A random-effect meta-analysis was performed for pooling the prevalence of controlled hypertension. As shown in Fig. (**2**), the overall prevalence rate of controlled hypertension was 36.79% (95%CI 29.06, 45.26). The sensitivity analysis for this meta-analysis is shown in Supplementary Fig. (**S1**). Sorted by proportion, the deletion of Binaqeel *et al.* [20] resulted in a prevalence rate of 34.0% (95%CI 28.0, 41.0), while leaving Berraho *et al.* [7], which resulted in a 39.0% (95%CI 31.0, 47.0) prevalence rate. Moreover, as shown in Supplementary Fig. (**2**), there is no apparent asymmetry in the funnel plot similar to Egger’s test, which was indicative of no publication bias (*p*-value 0.2).

#### 3.2.1 Subgroup Analysis Based on Study Design

Fig. (**2**) represents the prevalence rate based on study design. The case-control (one study) subgroup had a prevalence of 71.02% (95% CI 66.43, 75.31); the inclusion of only one study made it not feasible to conduct the I2 heterogeneity test. Cross-sectional studies, comprising 12 studies involving 8,225 patients, yielded a pooled prevalence of 33.94% (95% CI 23.89, 45.67), with an I^2^ heterogeneity of 99% under a random-effect model. In contrast, the meta-analysis of cohort studies, including seven studies with 20,103 individuals, estimated a prevalence rate of 37.50% (95% CI 30.36, 45.23) and an I^2^ heterogeneity of 98% in a random-effect model. The random effects model for all studies combined shows an overall prevalence of 36.79% (95% CI 29.06, 45.26). The heterogeneity across all studies was high, with I^2^ = 99% and τ2 = 0.6601 (*p* < 0.01), indicating substantial variability between studies. Significant differences between subgroups were observed, as evidenced by the test for subgroup differences in the random effects model (χ2 = 70.09, df = 2, *p* < 0.01). The prevalence rates varied widely across individual studies, ranging from 10.51% to 83.07%. The studies with the largest sample sizes had prevalence rates of 42.84% (95% CI 42.27, 43.41) and 36.79% (95% CI 29.06, 45.26), respectively. These findings highlight the considerable variability between studies and subgroups and the differences in prevalence estimates under the random effects model.

#### 3.2.2 Subgroup Analysis based on BP Target

As illustrated in Fig. (**3**), three cutoffs were used for target-controlled BP among the studies. Subgroup analysis based on these targets shows that the overall observed prevalence of controlled BP by ≤140/90 mmHg cutoff was 36.48% (95%CI 27.12, 46.97), and I^2^: 98% in a random-effect model of 18474 data. In the ≤130/80 mmHg cutoff subgroup, the prevalence was 38.18% (95%CI 24.48, 54.06), and I^2^: 99% in a random-effect model of 10066 data. Finally, in one study investigating the BP target of 130/85 mmHg (22), the prevalence rate was 30.14% (95%CI 24.01, 36.86).

#### 3.2.3 Subgroup Analysis Based on Gender

Five studies reported the BP control in diabetic hypertensive patients among men and women separately (19, 26-29). Meta-analysis of the men subgroup resulted in a prevalence of 43.51% (95%CI 20.03, 70.31) and I^2^: 98% in a random-effect model of 1732 data for BP control. However, women had an overall prevalence of 53.23% (95%CI 36.08, 69.64), and I^2^: 98% in a random-effect model of 2215 data. This analysis is shown as a forest plot in Fig. (**4**).

#### 3.2.4 Subgroup Analysis Based on Country

Among three studies conducted for each country, the prevalence rate was 26.14% (95%CI 21.79, 31.01), I^2^: 44% for Pakistan (13, 14, 19), 50.72% (95%CI 22.75, 78.25), I^2^:100% for Saudia Arabia (18, 20, 23), 35.12% (95%CI 23.65, 48.61), I^2^: 97% for Iran (21, 24, 28), 47.90% (95%CI 44.54, 51.29), I^2^:78% for Jordan (26, 27, 29), and 35.50% (95%CI 22.96, 50.40), I^2^: 99% for Oman (15, 16, 22). Details of this analysis are presented in Fig. (**5**).

#### 3.2.5 Meta-regression

Supplementary Table **S2** illustrates the impact of various confounders, including gender, BP target, type of study, and type of diabetes, on the prevalence rate of controlled hypertension. The meta-regression analysis revealed the following results: The BP target of 140/90 mm Hg, compared to the reference target of 130/80 mm Hg, showed a significant reduction effect on the prevalence rate of controlled hypertension, with an estimate of -0.62 (95% CI: -1.21, -0.02, *P* = 0.042). In contrast, the BP target of 130/85 mm Hg did not show a significant effect, with an estimate of -0.53 (95% CI: -2.32, 1.26, *P* = 0.560). When considering the reference category of the total population, the female subgroup had an estimated 0.79 (95% CI: -0.04, 1.61, *P* = 0.062), which approached statistical significance. The male subgroup had an estimate of 0.41 (95% CI: -0.42, 1.24, *P* = 0.335), which was not statistically significant. In comparison to cohort studies, case-control studies showed an estimate of 1.38 (95% CI: -0.56, 3.32, *P* = 0.164), while cross-sectional studies had an estimate of 0.02 (95% CI: -0.88, 0.93, *P* = 0.957). Neither type showed a significant impact on the prevalence rate of controlled hypertension. Comparing type 1 and type 2 diabetes combined (T1DM & T2DM) to type 2 diabetes mellitus (T2DM) alone, the estimate was 0.45 (95% CI: -0.90, 1.80, *P* = 0.514), indicating no significant difference. These findings underscore the significance of the BP target of 140/90 mm Hg in reducing the prevalence rate of controlled hypertension, while other variables did not demonstrate a statistically significant impact.

## 4 DISCUSSION

This systematic review and meta-analysis, the first to assess controlled hypertension prevalence in hypertensive diabetic patients in the EMR region, unveils a sub-optimal overall control rate of 36.79% (95% CI 29.06, 45.26). Our subgroup analyses reveal significant variations in BP control across different study designs, BP targets, and countries, with the meta-regression underscoring the importance of a 140/90 mm Hg BP target in significantly reducing hypertension rates. Notably, women demonstrate better BP control than men, highlighting the necessity for targeted strategies in this group. These findings and the heterogeneity observed underscore the complexity of managing this condition in the EMR region and could have significant public health implications. The coexistence of arterial hypertension and diabetes represents a detrimental combination for cardiovascular health. Effective control of blood pressure and blood glucose is essential in these patients to reduce the risk of cardiovascular and other diabetes-related complications. The major benefit of blood pressure control in diabetes is the significant reduction in cardiovascular and renal endpoints [31, 32]. Recent meta-analyses have confirmed the positive impact of blood pressure lowering in diabetic patients, showing that for every ten mmHg reduction in systolic blood pressure, there is a 13% reduction in mortality risk, an 11% reduction in the risk of cardiovascular events, a 12% reduction in the risk of coronary heart disease, and a 27% reduction in the risk of stroke [33]. These findings underscore the importance of achieving and maintaining target blood pressure values to maximize cardiovascular and renal protection in diabetic patients. Additionally, blood pressure control has been suggested to improve microvascular complications during diabetes, particularly microalbuminuria or diabetic retinopathy. However, the evidence for these effects is less robust than for macrovascular disease [34]. The prevalence of hypertension has been suggested to be twice as high in patients with diabetes compared to those without [35]. Additionally, isolated systolic hypertension resistant to treatment has been shown to be more common in diabetic patients [36]. In the European Action on Secondary Prevention through Intervention to Reduce Events (EUROASPIRE) IV study that was conducted in 24 countries across Europe with 6187 diabetic patients, almost half of the patients (54%) achieved the blood pressure target of less than 140/90 mmHg [37]. This is higher than our subgroup of ≤140/90 mmHg, which was 36.48% (95%CI 27.12, 46.97). The differences in achieving hypertension control, as seen between the outcomes of the EUROASPIRE IV study and regions like the Eastern Mediterranean, could largely be attributed to the educational programs and infrastructure in European countries. High-income European nations have developed advanced healthcare systems, evidenced by their ability to attain treatment rates of up to 80% and control rates of up to 60% [38]. This success is underpinned by efficient healthcare infrastructure, including universal health coverage and established primary care systems, which are less prevalent in lower-income regions. Effective educational and interventional strategies, such as employing non-physician health workers, patient follow-ups, and technology-assisted reminders, have played a crucial role. Moreover, cultural, and socioeconomic factors, including dietary improvements and higher overall socioeconomic status, contribute significantly to better health outcomes. However, it is challenging to determine whether the reduction in blood pressure (BP) is a primary effect of the diet, such as the Mediterranean diet (MedDiet), or a secondary effect resulting from overall metabolic health improvement. For instance, moderate weight loss, often associated with healthier dietary practices, is strongly correlated with decreased BP. Distinguishing the primary effects of specific food components from secondary metabolic effects requires careful study design that controls for confounders such as weight loss, caloric restriction, and an overall healthier lifestyle. The Hameed *et al.* study emphasizes the need for research that meticulously separates these factors to better understand the direct impact of dietary components on BP. They suggest that future studies should focus on hemodynamic changes that occur with MedDiet and other plant-based diets, considering these confounders to isolate the true effects of the diet on BP [39]. Ventriglio *et al.* highlighted that MedDiet is effective in controlling hypertension due to its rich content of fruits, vegetables, whole grains, and healthy fats, particularly olive oil. They noted that these components contribute to improved endothelial function and reduced arterial stiffness, key factors in lowering BP. Additionally, the anti-inflammatory and antioxidant properties of the MedDiet play a significant role in managing hypertension, further distinguishing it from other dietary patterns. Moreover, the MedDiet promotes a favorable metabolic and cardiovascular balance, enhancing insulin sensitivity and reducing oxidative stress [40]. These combined effects help maintain overall cardiovascular health, making implementing MedDiet a comprehensive approach to hypertension management. In addition, studies have shown that molecular changes in the blood vessel walls, particularly in conditions of oxidative damage, can significantly impact cardiovascular health. Research involving postmenopausal rats fed with repeatedly heated palm oil revealed that hypercholesterolemia, increased lipid peroxidation, and hyperhomocysteinemia contribute to the pathogenesis of atherosclerosis. The ultrastructural examination of these rats’ aorta indicated a pronounced endothelial layer disruption, especially with five-times-heated palm oil. This disruption was associated with oxidative damage caused by lipid peroxidation products generated during the repeated heating process. These findings suggest that consuming oxidized fats, often resulting from faulty diets, can exacerbate vascular damage and accelerate atherosclerotic changes, particularly in estrogen-deficient conditions [34]. Furthermore, Regular health checks and screening programs in Europe lead to early detection and management of hypertension, a systematic approach not as widespread in lower-income areas. The availability and affordability of a wide range of antihypertensive medications in high-income countries further enhance treatment efficacy. Additionally, expanding universal health coverage in European countries has been pivotal in improving hypertension care and incorporating guidelines, training, and essential equipment in healthcare facilities. Finally, the variation in real-world effectiveness of treatment in these countries reflects robust health-system features that enable high-quality care and diverse pharmacological approaches. In contrast, lower-income regions struggle with these aspects, highlighting their multifaceted challenges, including infrastructure, education, socioeconomic factors, and healthcare system efficiency [41, 42]. Regarding the difference between countries in the EMR region, Somalia has the highest prevalence in the region (26.4%), and the United Arab Emirates (UAE) has the lowest (14.7%) [42]. On the other hand, the prevalence of diabetes was the highest among all the WHO regions, with Kuwait, Qatar, and Saudi Arabia, being among the top 10 countries in terms of prevalence which could be due to unhealthy dietary behaviors, low physical activity, and multiparity [42-44]. There are expectations for a doubling in diabetes incidence in EMR region countries, with four out of ten diabetic patients not yet diagnosed [42]. Our study also showed that only 36.79% (95% CI 29.06, 45.26) of known diabetic cases with hypertension had controlled their BP, and there might be more unknown cases of diabetes not being aware of their hypertension as well. These could result in a higher rate of adverse outcomes from different conditions, mainly cardiovascular (CV) ones. A deeper look at these numbers and comparing them with existing literature reveals important findings. The UAE, which has a lower prevalence of hypertension, has been implementing the Weqaya Screening Program. The effectiveness of this initiative is supported by these low prevalence numbers. On the other hand, our findings indicate that Jordan with 47.90% (95% CI 44.54, 51.29) has a higher prevalence of controlled hypertension in diabetic patients, which is consistent with the high medication rates reported in the region by existing literature. These observations suggest that specific healthcare programs, like the Weqaya Screening Program in the UAE, are effectively managing hypertension, particularly in patients with diabetes. Additionally, the correlation between higher medication rates and better hypertension control in Jordan highlights the importance of medication adherence and access in managing such health conditions. As for the importance of controlled hypertension in diabetes patients, there is a complex association between hypertension control and diabetes. It has been suggested that hypertension control can affect hyperinsulinemia and the risk of insulin-resistant states such as diabetes mellitus [45]. Among the studies, the Anglo-Scandinavian Cardiac Outcomes Trial reported that for a 10 mm Hg increase in systolic blood pressure (SBP), there is a 6% increase in the incident risk of diabetes mellitus [46]. In the CASE-J trial conducted in Japan, pulse pressure was shown to have an association with incident diabetes mellitus [47]. The co-existence of diabetes mellitus (DM) and hypertension markedly increases the risk for both macrovascular and microvascular complications. This increased risk extends to coronary heart disease, peripheral artery disease, stroke, retinopathy, and nephropathy, as well as to complications like left ventricular hypertrophy and congestive heart failure, more so than with either DM or hypertension alone. Clinical evidence underscores that effectively controlling BP substantially mitigates these risks in individuals with diabetes [48]. However, achieving optimal blood pressure targets remains a challenge for many patients with concurrent DM and hypertension. This challenge can often be attributed to the distinct pathophysiological aspects of DM, delays in treatment modification, and patient-specific factors such as suboptimal adherence to medication and limited access to specialized care. These insights emphasize the critical need for an integrated and proactive approach in managing both DM and hypertension, highlighting the importance of regular monitoring, patient education, and tailored therapeutic strategies to reduce the compounded risk associated with these coexisting conditions [49-53]. There is controversy about defining BP targets in patients with diabetes. For many years, the used target in common practice was ≤130/80 mmHg in non-proteinuric diabetic patients based on the UKPDS 38 [54], the Hypertension Optimal Treatment (HOT) study [55], and the Action in Diabetes and Vascular disease Controlled Evaluation (ADVANCE) trial [56] . However, no benefit or harm was shown in achieving these lower targets [57-59]. A meta-analysis of 49 trials investigating the effect of antihypertensive medications at different BP levels indicated that at BP levels higher than 140 mmHg, a decrease in BP resulted in a decline in all-cause and CV mortality [60] . On the other hand, lowering BP in those with baseline BP of less than 140 mmHg led to increased CV mortality. Similar findings were observed in another meta-analysis emphasizing that there is no benefit in achieving lower BP levels in diabetic patients compared to standards [61]. In our meta-analysis, most studies used ≤140/90 mmHg target in their analyses. Our study presents multiple policy implications, particularly in hypertension management among diabetic patients. Given the elevated risk of hypertension in this demographic, there should be an increased frequency of screenings for high BP in diabetic individuals. Concurrently, policymakers need to focus on strategies for effectively controlling hypertension in these patients. This approach's critical components include conducting educational sessions, prescribing suitable BP-lowering medications, and monitoring patient compliance. Despite providing a pooled estimate for controlled BP status in diabetic patients with hypertension, our study has several limitations that need to be mentioned. First, all meta-analyses had substantial heterogeneity, which was unresolved even after subgroup analysis. This limitation of our study might stem from the different settings in which the studies were performed. Second, a limited number of studies reported the results in males and females separately. Hence, our results for each gender might be limited. Finally, there was no data available for some of the countries in the region, which could threaten the generalizability of our findings to the whole EMR region.

## CONCLUSION

Our findings indicate that BP control among hypertensive diabetic patients in the EMR countries is sub-optimal, with only 36.79% (95%CI 29.06, 45.26). This information should serve as a directive for clinicians and policymakers to develop targeted policies and interventions aimed at improving BP control rates in this high-risk group.

## Figures and Tables

**Fig. (2):**
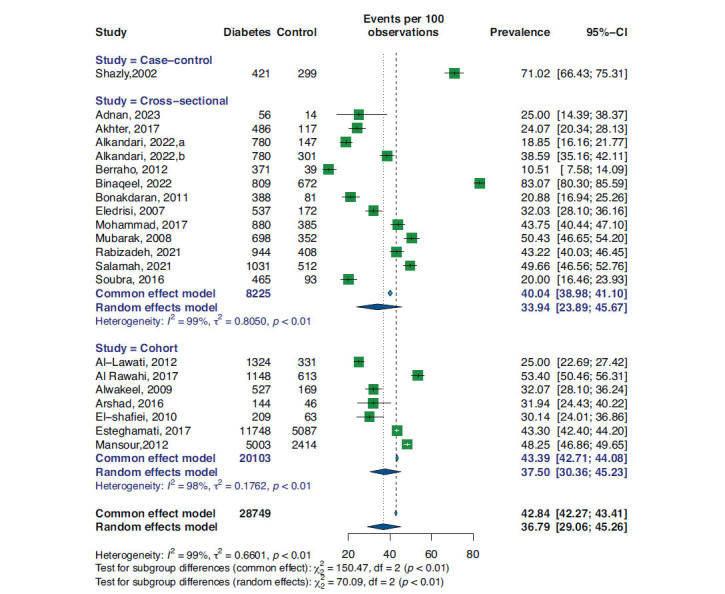
Forest plot and subgroup analysis based on study design for prevalence of BP control in diabetic patients.

**Fig. (3):**
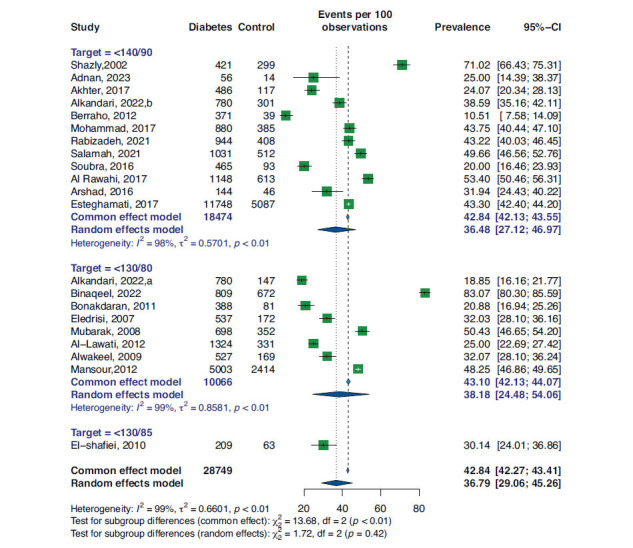
Forest plot and subgroup analysis based on BP target for prevalence of BP control in diabetic patients.

**Fig. (4):**
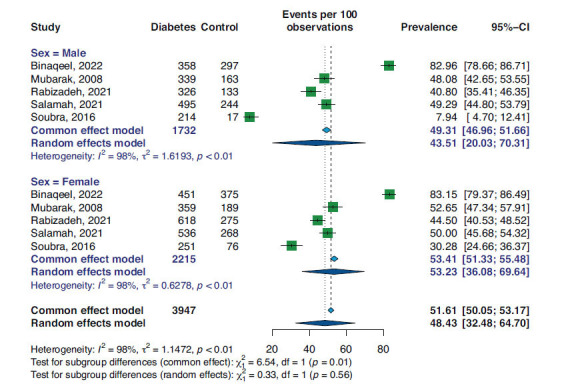
Subgroup analysis based on gender for prevalence of BP control in diabetic patients.

**Fig. (5):**
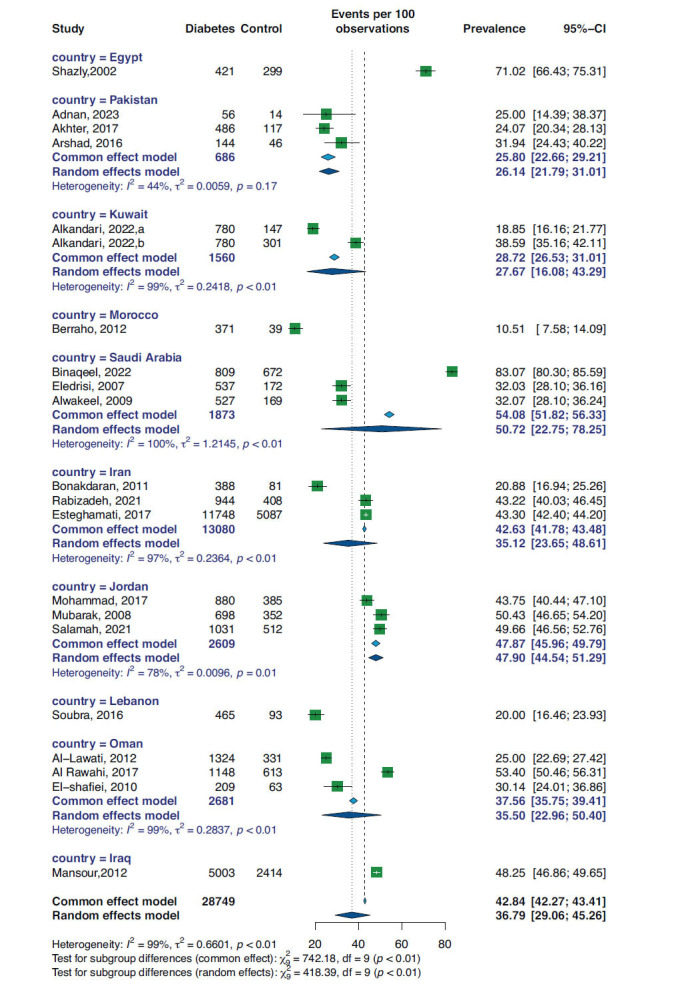
Forest plot and subgroup analysis based on the country for the prevalence of BP control in diabetic patients.

**Table 1:** Baseline characteristics of included studies.

**Study**	**City,** **Country**	**Study Design**	**Mean Age (Years)**	**Sex (Male %)**	**Cut-off Point (mmHg)**	**Hypertensive Sample Size (Controlled/** **Un-Controlled)**	**Prevalence Controlled %**	**95% CI of Prevalence Controlled (%)**	**Quality Assessment**
Adnan *et al.* (2023)	Lahore, Pakistan	Cross-sectional	49.0±10.7	36.9	≥140/90	42 (14/28)	25	14.39-38.37	High
Akhter *et al.* (2017)	Karachi, Pakistan	Cross-sectional	53.1± 11.9	51.8	>140/90	486 (117/369)	24.07	20.34-28.13	High
Al Rawahi *et al.* (2017)	Al Dakhiliyah Governorate, Oman	Retrospective Cohort	54.5±11.4	36	≥140/90	1148 (613/535)	53.4	50.46-56.31	High
Alkandari *et al.* (2022)	Six governates, Kuwait	Cross-sectional	50.1±10.7	58.9	>130/80	780 (147/633)	18.85	16.16-21.77	High
					>140/90	780 (301/479)	38.59	35.16-42.11	
Alwakeel *et al.* (2009)	Riyadh, Saudi Arabia	Cohort	66.9 ± 11.4	47	>130/80	527 (169/358)	32.07	28.1-36.24	High
Al-Lawati *et al.* (2012)	8 regions, Oman	Cohort	54±13	47	≥130/80	1324 (331/993)	25	22.69-27.42	High
Arshad *et al.* (2016)	Jammu and Kashmir, Pakistan	Prospective Cohort	51.42±10.93	59.94	>140/90	144 (46/98)	31.94	24.43-40.22	High
Berraho *et al.* (2012)	Fez, Sale and Taounate, Morocco	Cross-sectional	NA	31.3	≥140/90	371 (39/332)	10.51	7.58-14.09	High
Binaqeel *et al.* (2022)	Jeddah, Saudi Arabia	Cross-sectional	66.38± 10.8	44.25	≥130/80	809 (679/137)	T:83.07 M: 82.96 F:83.15	T: 80.3-85.59	High
Bonakdaran *et al.* (2011)	Mashhad, Iran	Cross-sectional	52.7±10.5	47.2	>140/90	388 (81/307)	21	16.94-25.26	High
Eledrisi *et al.* (2007)	Eastern and western provinces, Saudi Arabia	Cross-sectional	55.0± 12.6	45.3	>130/80	537 (172/365)	32.03	28.10-36.16	High
El-Shazly *et al.* (2002)	Alexandria, Egypt	Case-control	30->60	50.9	>140/90	421 (299/122)	71.02	66.43-75.31	High
El-Shafie *et al.* (2010)	Muscat, Oman	Retrospective Cohort	53.7±9.1	28.6	>130/85	209 (63/146)	30.14	24.01- 36.85	High
Esteghamati *et al.* (2017)	Iran	Prospective Cohort	59(M) 57(F)	33.5	>140/90	11749 (5087/6662)	43.3	42.5- 44.3	High
Mansour *et al.* (2012)	Basrah, Iraq	Prospective Cohort	51.4 ± 13.7	44.4	≥140/90	5003 (2414/2589)	48.2	46.86-49.65	High
Mohammad *et al.* (2017)	Amman, Jordan	Cross-sectional	60 ± 10.41	47	≥140/90	880 (385/495)	T: 43.75	T: 40.44-47.1	Low
Mubarak *et al.* (2008)	Amman, Jordan	Cross-sectional	NR	49.5	≥130/80	698 (352/346)	T: 50.43 F: 52.65 M: 48.08	T: 46.65-54.2	High
Rabizadeh *et al.* (2021)	Tehran, Iran	Cross-sectional	59.2±9.8	34.5	>140/90	944 (408/536)	T: 43.22 F: 44.5 M: 40.8	T: 40.03-46.45	High
Salameh *et al.* (2021)	Amman, Jordan	Cross-sectional	62.7±9.7	48	≥140/90	1031 (512/519)	T: 49.66 F: 50.0 M: 49.29	T: 46.56-52.76	High
Soubra *et al.* (2016)	Beirut, Lebanon	Cross-sectional	61.4±11.1	54	≥140/90	465 (93/372)	T: 20.0 F: 30.28 M: 7.94	T: 16.46-23.93	High

## Data Availability

The data supporting this study's findings are available from the corresponding author upon reasonable request.

## LIST OF ABBREVIATIONS

ADVANCE: Action in Diabetes and Vascular Disease Controlled Evaluation
BP: Blood Pressure
CI: Confidence Interval
CV: Cardiovascular
DM: Diabetes Mellitus
EMR: Eastern Mediterranean Region
EUROASPIRE: European Action on Secondary Prevention through Intervention to Reduce Events
HOT: Hypertension Optimal Treatment
PRISMA: Preferred Reporting Item for Systematic Reviews and Meta-Analysis
PROSPERO: International Prospective Register of Systematic Reviews
SBP: Systolic Blood Pressure
UAE: United Arab Emirates
UKPDS: United Kingdom Prospective Diabetes Study
WHO: World Health Organization

## STANDARDS OF REPORTING

STROBE and PRISMA guidelines have been followed in this study.

## SUPPLEMENTARY MATERIAL

Supplementary material is available on the publisher’s website along with the published article. The PRISMA and modified STROBE checklists are available as supplementary materials on the publisher's website along with the published article.
